# Bone marrow osteoma of the tibia: A case report

**DOI:** 10.3892/ol.2014.2564

**Published:** 2014-09-25

**Authors:** BEN-GEN ZHOU, MEI-YUAN LIU, LI-CHUN LV, HONG XIA

**Affiliations:** 1 Department of Orthopaedics, Guangzhou General Hospital of Guangzhou Military Command, Guangzhou, Guangdong 510010, P.R. China; 2 Department of Orthopedics, 458th Hospital of PLA, Guangzhou, Guangdong 510602, P.R. China; 3 Affiliated Tumor Hospital of Guangzhou Medical College, Guangzhou, Guangdong 510260, P.R. China; 4 Department of Pathology, 458th Hospital of PLA, Guangzhou, Guangdong 510600, P.R. China

**Keywords:** osteoma, tibia, excision, lesion

## Abstract

In this study, an unusual case of osteoma is presented, whereby a bone marrow osteoma was identified in the tibia. No previous cases of bone marrow osteoma have been reported. In this case, an eight-year-old male presented with discontinuous discomfort in the right distal calf for six months. Radiological examination and computed tomography revealed a radiopaque lesion within the affected bone. A technetium-99m bone scan revealed focally increased uptake in the same region. Together, these observations prior to surgery indicated that the patient may suffer from bone disease. Subsequently, a surgical excision was performed and the biopsy specimen was identified as bone marrow osteoma. Following surgery, the symptoms were eradicated and the prognosis was positive during the 24-month follow-up period. Bone marrow osteoma should be considered when a patient suffers from discontinuous and unexplained limb discomfort.

## Introduction

Osteoma is a rare but benign tumor, which is caused by abnormal growth of bone or excessive proliferation of other tissues. Microscopically, osteoma consists of compact and spongy bone. Osteoma exhibits continuous growth during childhood rather than exhibiting growth cessation during adulthood. This is the major feature that distinguishes osteoma from other bony exostosis, including tori (1–4). During their slow and steady increase in size, osteomas remains asymptomatic until they reach a size that is sufficient to cause disfigurement and/or direct interference with the normal function of its anatomical location. Excision of osteoma is often unnecessary, however, surgery is required in the presence of apparent symptoms.

Various theories have been suggested to explain the pathological mechanism of osteoma. For example, these lesions have been associated with the abnormal enlargement of the fetal periosteum or residual cartilage, reactive lesions due to trauma, muscle traction and infection, as well as true neoplasms. However, a specific cause-effect association is difficult to establish (5–7).

In this study, a case of a patient with bone marrow osteoma located in tibia who underwent surgical excision of the lesion is presented. This study was approved by the ethics committee of Guangzhou General Hospital of Guangzhou Military Command (Guangzhou, China) and written informed consent was obtained from the patient’s family.

## Case report

**Figure 1:**
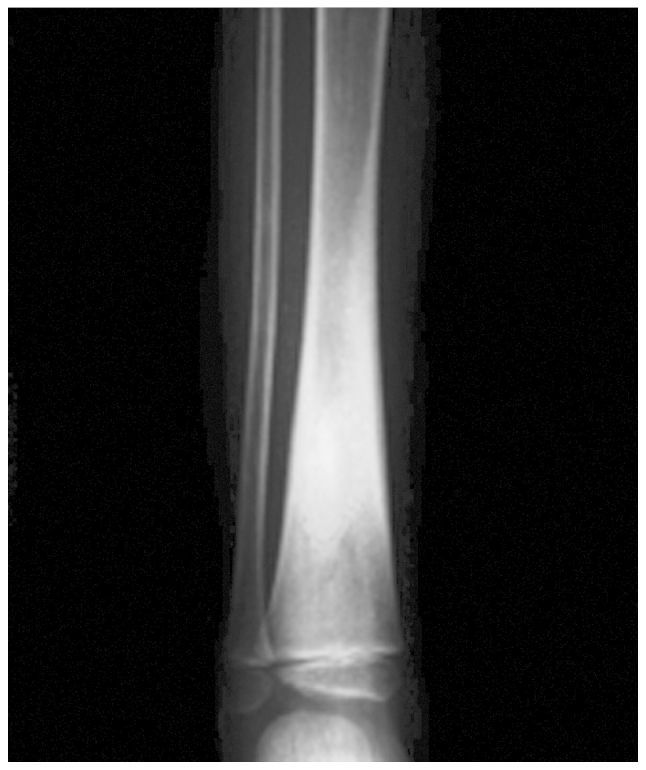
Anteroposterior radiography showed increased radiopaque nidus in the distal tibia.

**Figure 2:**
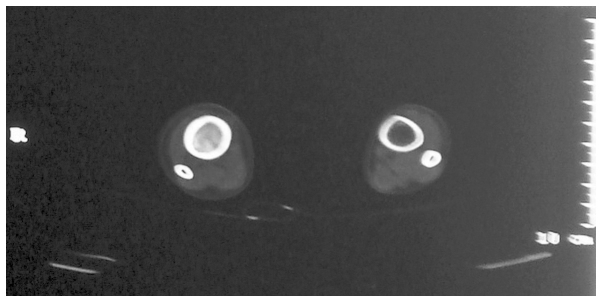
Computed tomography of the tibia showed a narrow medullary cavity packed with high density osteoid tissues.

**Figure 3:**
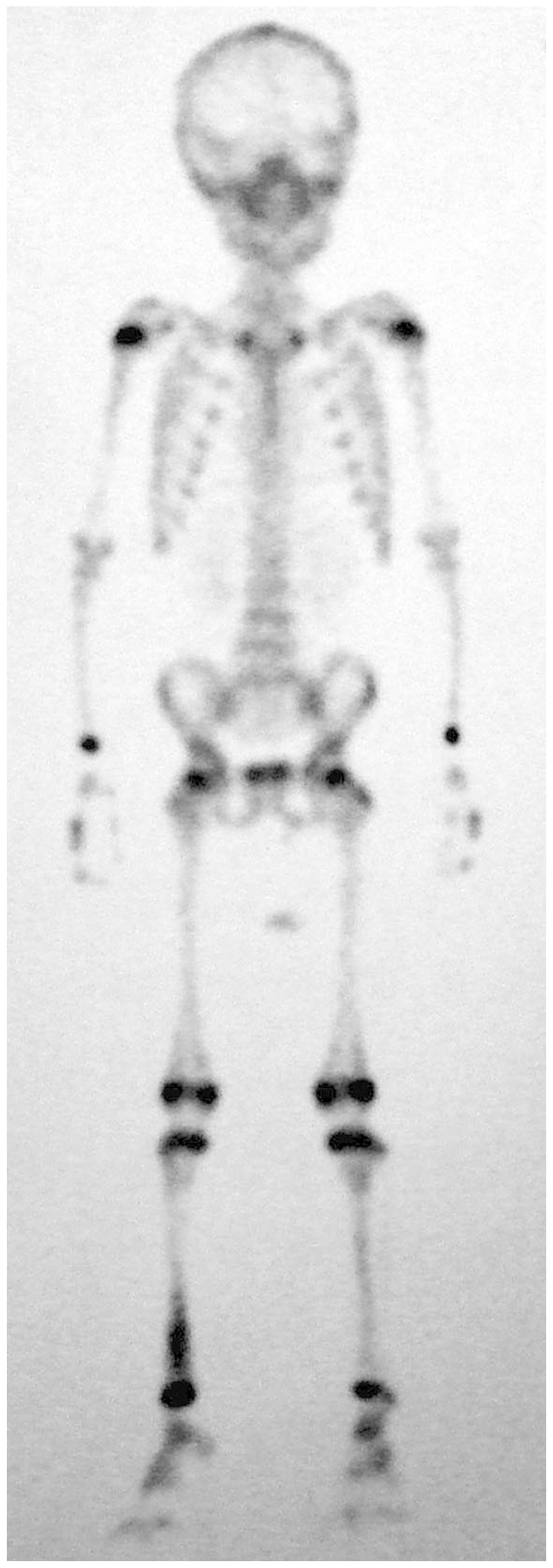
Technetium-99m bone scan showed focally increased uptake in the distal tibia.

An eight-year-old male was referred to the 458th Hospital of PLA (Guangzhou, China). The patient had complained of discontinuous discomfort in the right distal calf for six months. The discomfort worsened at night and was not improved by aspirin administration. Physical examination was normal with the exception of a pressing pain at the distal calf. Plain radiographies showed increased bony density in the distal tibia (Fig. 1). Computed tomography (CT) scans of the distal tibia revealed that medullary cavity was narrow and packed with high-density osteoid tissue (Fig. 2). A technetium-99m bone scan showed focally increased uptake in the same region (Fig. 3). Collectively, these observations indicated that the patient may have suffered from bone disease.

**Figure 4:**
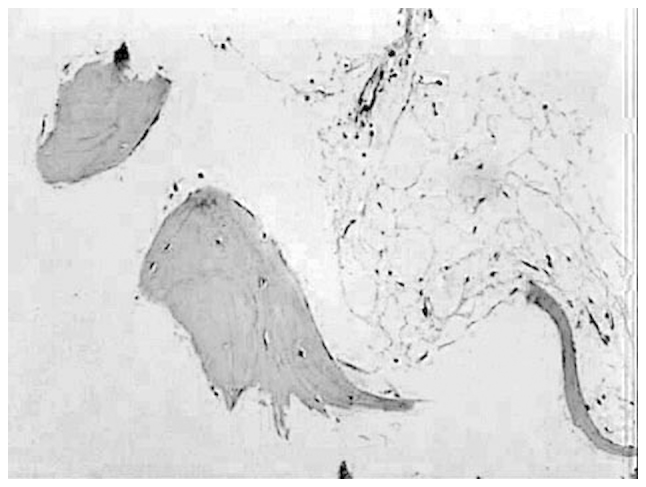
The excised mass shows a disordered trabeculae structure composed of mature bone. Sparse fiber, vessels and adipose tissues were identified surrounding the bone trabecula, but without osteoblasts. Hematoxylin and eosin stain; magnification, ×40.

The patient subsequently underwent surgical excision of the lesion. The lesion size was measured according to the preoperative X-ray and CT results. The lesion site with an 3 cm diameter, marginally larger than the preoperative measurements, was opened at the middle of tibia cortex. Surgical findings revealed a hard bone substance that had formed in the medullary cavity. A small section of diseased tissue was subsequently subjected to intraoperative frozen sectioning for pathological examination. The examination results indicated the presence of a benign tumor. The lesion was then completely removed using a spatula and power drill. Histological examination revealed that the excised mass was a bone marrow osteoma. (Fig. 4). Following surgery, the symptoms were eradicated. The patient was followed up for 24 months without any recurrence.

## Discussion

Osteoma often occurs in the skull and facial bones, however, it may also occur in the limbs and other body parts. Based on its location, osteoma is divided into peripheral, central and extraskeletal osteomas. Peripheral osteomas arise from the cortical plate, whereas central osteomas develop as masses on endosteal bone surfaces and extraskeletal osteomas have rarely been reported (8,9). Osteoma in the bone marrow cavity is termed bone marrow osteoma. Various studies have reported that osteomas may exist in multiple unusual regions, including the acoustic meatus (10), middle ear ossicles (11) and false vocal folds (12). Notably, whether osteoma may occur in the bone marrow remains unclear, however, this study presented the case of a patient with bone marrow osteoma located in the tibia that underwent surgical excision of the lesion.

The primary differential diagnosis includes other radiopaque masses, including osteoblastoma, osteoid osteoma, fibro-osseous lesions, cementoblastoma, osteosarcoma, exostosis, complex odontoma, sessile osteochondroma and end-stage osteomyelitis (13,14).

Patients with osteoblastoma or osteoid osteoma usually exhibit a medical history of pain, a characteristic clinical symptom, which is not often directly associated with osteomas. Furthermore, the pain caused by an osteoid osteoma is distinguished symptomatically from osteoma and osteoblastoma by the presence of a history of pain ameliorated by non-steroidal anti-inflammatory drugs. In addition, osteoblastomas and osteoid osteomas grow more rapidly than osteomas (15). Radiographical features of an osteoblastoma or osteoid osteoma generally exhibit a round or oval well-delineated radiolucent defect. However, an osteoma exhibits a round or oval well-circumscribed radiopaque mass with a broad base (16). An osteoid osteoma is further distinguished radiographically from osteomas and osteoblastoma in the presence of a distinct rim of sclerosis, as well as an identifiable radiopaque nidus (16). Microscopically, osteoblastoma and osteoid osteoma exhibit abundant osteoid trabeculae anastomosing in a loose fibrovascular connective stroma. The osteoid trabeculae in osteoblastoma and osteoid osteoma usually exhibits prominent osteoblastic rimming and a characteristic basophilic appearance. Osteoclastic giant cells are also often present in osteoblastoma and osteoid osteoma in addition to extravasated red cells, however, these latter features are absent in osteomas (17). By contrast, osteoma resembles normal compact or cancellous bone with variable amounts of fibrofatty bone marrow.

The clinical symptom of fibro-osseous lesions is painless swelling, which is similar to osteoma. On radiological examination, early fibro-osseous lesions are radiolucent, however, as they progress they appear as ill-defined ground-glass opacifications. In addition, for the majority of fibro-osseous lesions, growth is stabilized or restrained following the growth period, which allows them to be distinguished from osteoma (18).

Cementoblastoma may be excluded as an option if the lesion is not connected to the tooth at the time of diagnosis (19). Osteosarcomas grow rapidly and exhibit high recurrence rates, occurring preferentially in young adults aged between 10 and 20 years old, and the serum alkaline phosphatase level is elevated (20).

Exostosis is a hamartoma that grows in specific areas, including the lingual and buccal regions of the mandible, midline of hard palate, and buccal and hard palate regions of the maxilla, and growth stops following puberty (21). A peripheral osteoma may be differentiated from an exostosis in accordance with an accurate medical history and clinical features, however, no histological differences have been identified (22). Exostosis occurs more commonly than osteoma. It is a bony growth in the lingual plate of the maxillary bones, which is usually symmetrical, well circumscribed and associated with inflammatory or traumatic phenomena. An exostosis usually stops growing following puberty, whereas osteomas exhibit independent growth (22,23).

Complex odontoma is a clearly circumscribed radiopaque mass, with a density greater than that of bone, and it is surrounded by a narrow radiolucent rim (24). In sessile osteochondroma, the cortex of the lesion merges imperceptibly with the cortex of the host bone (25).

This case of bone marrow osteoma identified in the distal tibia provides an example of atypical presentation. Successful diagnosis and differential diagnosis may be obtained through clinical appearance, plain radiography, CT scan, emission computed tomography and histopathological examination. In conclusion, a diagnosis of bone marrow osteoma should be considered when a patient exhibits discontinuous and unexplained limb discomfort.
